# Mitigation and community preparedness in anticipating tsunami disasters in Muara Batu, Aceh

**DOI:** 10.4102/jamba.v15i1.1542

**Published:** 2023-12-05

**Authors:** Wheny Utariningsih, Vera Novalia, T. Saifullah

**Affiliations:** 1 Faculty of Medicine, Malikussaleh University, Lhokseumawe, Indonesia; 2 Faculty of Economics, Malikussaleh University, Aceh Utara, Indonesia

**Keywords:** disaster, mitigations, preparedness, tsunami, warning system, Muara Batu, Aceh

## Abstract

The tsunami disaster in 2004 that caused thousands of causalities forced the community of Aceh to carry out mitigation and preparedness. This study aims to describe about the mitigation and preparedness of the community in Muara Batu, North Aceh, Indonesia, in the face of the tsunami disaster. Data collection techniques used are in-depth interviews, observation, questionnaire and documentation. The results showed that the tsunami disaster mitigation in Muara Batu District was as follows: (1) construction of breakwater by government and plantation of coconut trees by residents along the coast and (2) the provision of education about disasters has been carried out. Meanwhile, related to the preparedness of the community in dealing with the tsunami disaster, the following were concluded: (1) the preparedness of the community in dealing with the tsunami disaster was classified as ready; (2) the knowledge and attitude of the household community in dealing with the tsunami disaster is classified as ready; (3) the community policy in dealing with the tsunami disaster is low; (4) the community emergency response plan in dealing with the tsunami disaster is ready; (5) the tsunami warning system still uses a hereditary tradition of using sirens and loudspeakers in the *meunasah* and/or *mushola*; (6) the level of mobilisation of community resources is still low.

**Contribution:**

This study recommends strengthening mitigation and preparedness with periodic community training by the village or district government. This will increase and strengthen synergies and interconnections between communities, village and district governments in dealing with the tsunami disaster.

## Introduction

The tsunami disaster that occurred in 2004 in Aceh province has caused many casualties and property losses. Indonesia’s Disaster Information Data released by Badan Nasional Penanggulangan Bencana (BNPB) states that the victims of the 2004 tsunami in Aceh reached 250°000 people who died (BNPB 2021). On the positive side, the tsunami incident became an impetus for the government to improve disaster management in Indonesia.

*Undang-undang tentang penanggulangan bencana* or the law on disaster management explained that disaster management is a series of efforts that include the establishment of development policies that pose a risk of disaster, disaster prevention, emergency response and rehabilitation. Disaster management is basically an effort made to save the community from existing disaster threats by anticipating and reducing risks, coping in the event of a disaster and recovering its impacts.

Based on the risk assessment that has been carried out, North Aceh is one of the districts in Aceh Province with a moderate to high risk of a tsunami disaster (BNPB 2016). Therefore, it is still relevant to conduct research on mitigation and community preparedness on the coast of Aceh in dealing with the tsunami disaster. In this study, the location chosen was Muara Batu District, North Aceh, with a focus on four villages, namely Tanoh Anoe Village, Pante Gurah, Keude Mane and Cot Seurani. The reason is because in the 2004 tsunami disaster, Muara Batu sub-district was one of the sub-districts in North Aceh with a high number of fatalities and material losses. At least 700 victims died in the 2004 tsunami disaster (klikwarta.com 2019).

Preparedness as stated in Law no. 24 of 2007 can be understood as a series of activities carried out to anticipate disasters through organisation and through appropriate and efficient steps. Parameters in preparedness according to LIPI – United Nations Educational, Scientific and Cultural Organization (UNESCO) are knowledge and attitudes; policies and guidelines, plans for disaster emergencies; disaster warning system and resource mobilisation (LIPI-UNESCO/ISDR 2006).

Meanwhile, mitigation based on the definition of Coppola (2007) is an effort to reduce disaster risk through physical development as well as capacity building or awareness. There are two disaster mitigation, namely structural and non-structural mitigation. Structural disaster mitigation is a type of mitigation that deals with physical development and repair. One example of its activities is to build earthquake-resistant buildings in areas that are at high risk of earthquake disasters. Meanwhile, non-structural mitigation is a type of mitigation that focuses more on human behaviour. Activities that can be carried out include making regulations in which there is a spatial plan, controlling population density in disaster risk areas, increasing public awareness of disaster risk and other activities (Adiyoso 2018).

The tsunami disaster is one type of disaster that is destructive and deadly. Therefore, it is important for people who are in areas at high risk of tsunami to carry out disaster mitigation and have preparedness for a tsunami disaster.

Studies on disaster mitigation were carried out by Zulfa, Widyasamaratri and Kautsary (2022) and Dewi and Istiadi (2016) who stated that mitigation had been implemented by the community. In addition, related researches on preparedness, both community and school preparedness, mention that the level of preparedness in dealing with disaster was almost ready (Fathird & Desfandi 2022; Fitriyani, Emaliyawati & Mirwanti 2021; Mayzarah & Batmomolin 2021; Utariningsih et al. 2021). However, research on disaster mitigation and disaster preparedness is still limited, especially on the mitigation and preparedness of village governments in facing the tsunami disaster. Therefore, it is important to research tsunami disaster mitigation and preparedness that focuses on the community and village government. This is because the community and village government are directly dealing with tsunami disaster.

## Research methods and design

In this study, there are two discussions that become the focus of the study, namely about tsunami disaster mitigation and community preparedness in dealing with tsunami disasters in Muara Batu District, North Aceh. Data collection techniques used were in-depth interviews, observation and documentation. In-depth interviews were conducted on the government and questionnaires were given to the community of Tanoh Anoe, Pante Gurah, Keude Mane and Cot Seurani villages. In the village government, interviews were conducted on the headman (Keuchik), while for informants from the community, they were selected using the purposive sampling method because in qualitative research what was important was the accuracy of the informants and the completeness and integrity of the data with the research context. Observations were made to directly see disaster management efforts for pre-disaster as a support for research data, while the documentation is performed by collecting reading materials relating to the subject matter in this study.

### Ethical considerations

Ethical clearance to conduct this study was obtained from the University of Malikussaleh Health Research Ethics Committee (No. 040/KEPK/FKUNIMAL-RSUCM/2022).

## Results and discussion

### Disaster mitigation

#### Implementation of spatial planning, development arrangements and infrastructure development

The tsunami disaster that occurred in 2004 in Aceh has made the government aware of the importance of disaster management, especially for the North Aceh government. In tackling the tsunami disaster, the North Aceh government in its spatial plan, which was ratified in the North Aceh Regency *Qanun* or law Number 7 of 2013 concerning the North Aceh Regency Spatial Plan for 2012–2032 which discusses tsunami-prone areas, namely areas along the coast: Muara Batu District, Dewantara District, Syamtalira Bayu District, Samudera District, Tanah Pasir District, Lapang District, West Baktiya District, Seunuddon District and Tanah Jambo Aye District. The *Qanun* or Law also explains that the general provisions for zoning regulations for tsunami areas are as follows:

- Utilisation of space for supporting infrastructure in order to reduce disaster risk
- Limited and/or conditional use of tsunami-prone area space for agricultural, plantation, fishery and forest activities, with appropriate types of flora and fauna, appropriate land management technology, and support of natural structures and/or artificial structures to withstand tsunami waves
- Restrictions on buildings and settlements that have been built by applying the building standards (building code)
- Prohibition of the construction of important buildings such as industries or factories, public facilities and other buildings.

**FIGURE 1:**
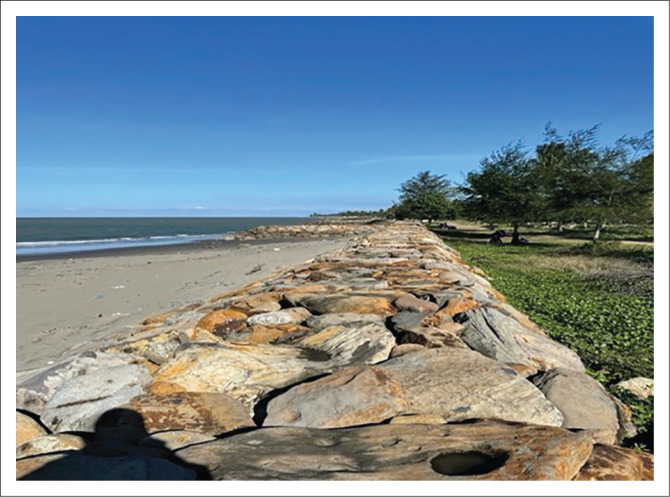
Breakwaters (*keroncong*) on the coast of the villages of Cot Seurani, Tanoh Anoe and Meunasah Lhok.

Based on the data obtained, on the coast of the villages of Cot Seurani, Tanoh Anoe and Meunasah Lhok, breakwaters (*keroncong*) have been built (Figure 1). However, the construction of breakwaters has not been fully developed along the coast of Muara Batu District, namely on the coast of Gampong Tanoh Anoe, Cot Seurani and Meunasah Lhok. The construction of this keroncong is constrained by costs. However, based on information from the sub-district government, the construction budget of breakwater and/or *keroncong* along the coast of Muara Batu has been proposed to the central government through the local government. For tsunami disaster mitigation, a seawall should be built, but the only thing in this area is a building to prevent erosion. As in the research conducted by Inabah et al. (2020), that seawalls along the Sunda Strait were not designed for tsunami disaster mitigation. The study also found that there was no seawall for tsunami disaster mitigation in Indonesia.

**FIGURE 2:**
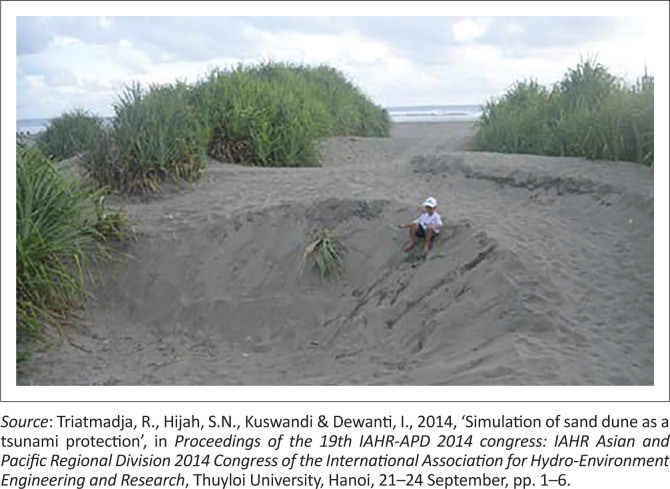
Sand dunes at Parangtritis Beach.

The construction of breakwaters is one of the government’s efforts to protect coastal areas. According Soviana et al. (2023), breakwater and/or embankment can reduce tsunami inundation level and protecting low-lying areas such as on the coast of Bnada Aceh City. In this case, the breakwater building is also used as a protection for the people on the coast when a tsunami disaster occurs. Another research also mentioned that one of the materials that is able to protect the coast from tsunamis is sand dunes (Figure 2). Triatmadja and Warniati (2021) stated that sand dunes are quite difficult to be breached by the tsunami. In the research to test the sand dunes in holding off the tsunami using physical and mathematical models by Triatmadja et al. (2014), it was found that the lower the sand dunes from the tsunami, the more damage level of the sand dunes would be. In the test that was carried out, it was seen that there were still quite a lot of sand dunes remaining and still surviving in protecting the downstream areas.

In addition to the construction of breakwaters, the spatial regulations in North Aceh instruct the limited and/or conditional use of tsunami-prone areas for agricultural, plantation, fishery and forest activities, with appropriate types of flora and fauna, appropriate land management technology, and support of natural structures and/or artificial structures to withstand tsunami waves. However, at the research site, several spots along the coast are used as tourist attractions. Along the coast of Tanoh Anoe, for example, there are many small food vendors lined up. In addition, the breakwater building is also used as a seat for local tourists.

**FIGURE 3:**
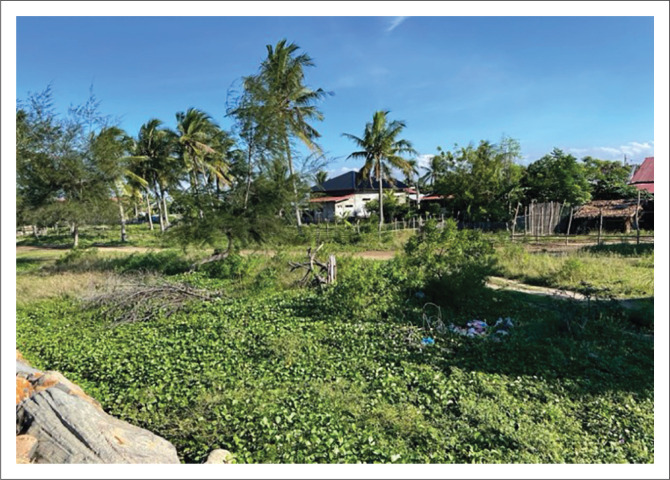
People’s houses in disaster-prone area.

The next rule related to spatial planning in North Aceh is the limitation of buildings and settlements that have been built by implementing the building code and prohibiting the construction of important buildings such as industries or factories, public facilities and other buildings. This rule is not enforced by some villages such as in the village of Tanoh Anoe. In the village of Tanoh Anoe, a fish auction place was built right on the beach. In addition to the fish auction place, many people’s houses are standing near the beach (Figure 3). The construction of settlements in tsunami-prone areas will certainly increase the level of vulnerability of these settlements. Moreover, the tsunami is one of the most destructive disasters and can occur at any time. The construction of this settlement is because many residents do not have other land to build houses or buildings. As stated by the village chief, ‘We already know the rules regarding the prohibition of development. The public also knows. But it can’t be helped, the community doesn’t have other land to build houses’ (Interview, 2021).

**FIGURE 4:**
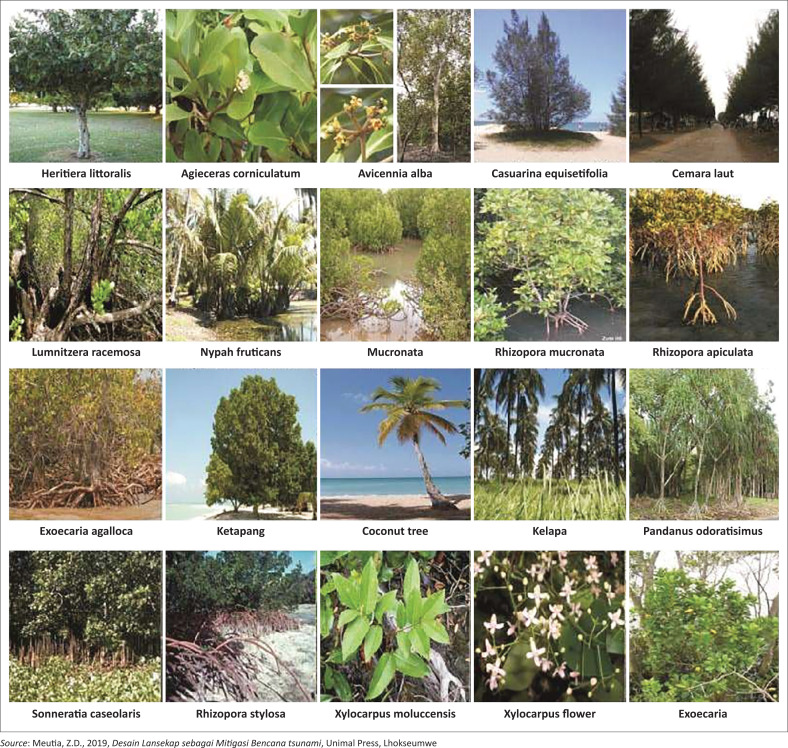
Coastal vegetation and mangrove type in Asia.

The construction of houses carried out is also not in accordance with building standards. Regarding building standards, many people and village governments do not know about it. Apart from building, the community also planted coconut trees along the coast. The planting of coconut trees will slow or restrain the waves when a tsunami occurs. Tanaka (2009) stated that coastal vegetation is a natural method to reduce tsunami waves. Some of the vegetations in Asian continent in an area that is directly bordering the sea are dominated by mangroves which are divided into two zones. The name of the zones are zone I, which consists of *Rhizophora mucronata* (Asiatic mangroves), *Rhizophora apiculata* (large-leaved orange mangroves), *Rhizophora stylosa* (red mangroves), *Sonneratia alba* (apple mangroves) and *Avicennia alba* (black mangroves), and zone II, which consists of *Rhizophora mucronata* (Asiatic mangroves), *Aegiceras corniculatum* (river mangrove), *Nypa fruticans* (nipah palm), *Sonneratia caseolaris* (crabapple mangroves), *Xylocarpus* spp. (cannonball mangroves), *Lumnitzera racemosa* (white-flowered black mangroves), *Heritiera littoralis* (tulip mangroves) and *Excoecaria agallocha* (blind-your-eyes mangroves) (Figure 4). Both of them can reduce the height of tsunami waves up to 50% and slow down the speed of tsunami waves (Meutia 2021).

**FIGURE 5:**
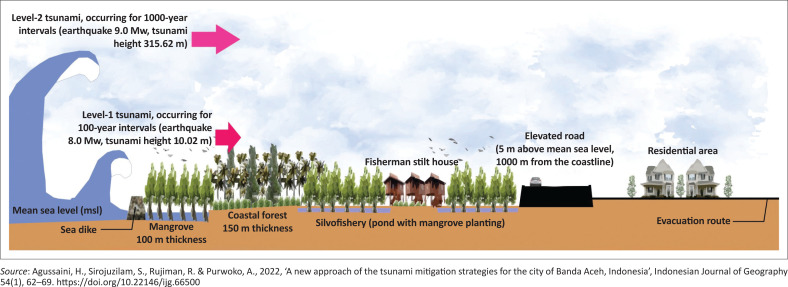
Multilayer tsunami defence system.

In the study conducted in Banda Aceh, Indonesia has implemented multilayer tsunami disaster defence system, Figure 5, namely to strengthen and maintain the existing wave breaking equipment and seawall; designate mangroves as coastal protected forests; convert ponds into silvofisheries; provide housing for fishermen with special requirements such as stilt houses; elevate the ring road to reduce potential losses; move settlements to low-risk areas for tsunami disasters and determine evacuation locations in safe zones (Agussaini et al. 2022).

#### Implementation of education, counselling and training

In Muara Batu sub-district, there has been a tsunami disaster that caused the death of more than 700 people. Many informants said that they were still traumatised by the incident. On the one hand, public knowledge about the tsunami disaster has increased. In the questionnaire given, many people know about the tsunami disaster, its causes and how to save themselves. This is because of the experience of the tsunami in 2004. The experience of this incident made people understand that the tsunami was a disaster caused by an earthquake. In addition, it is marked by the receding seawater.

This knowledge is not only obtained from experience but also the community has received training from non-governmental organisations that came after the tsunami disaster. The village chief of one village explained that: ‘After the tsunami disaster, they were taught about the tsunami disaster. If I’m not mistaken, the name is REKOMPAK. We are also given trauma healing’ (Participant 1, 40 years old, Village Head).

Knowledge is the result of providing education, socialisation and experience that will become capital for the community in responding to disasters. Socialisation regarding the tsunami disaster to the people of Muara Batu has not been carried out since 2004. Moreover, education at school also has not implemented a structured disaster-related curriculum. Implementation of disaster education is an important part of every plan to mitigate the risk of the disaster, which aims to increase public awareness regarding disaster prevention and reduction with educational programmes appropriate to the community and in different school environments (Tan et al. 2016). Result of the study conducted by Ramadhani, Miladan and Kusumasti (2023) showed that the level of non-structural tsunami disaster mitigation in Kuta District, Bali, Indonesia, is also in the poor category.

Basically, related to education in schools, the government has prepared regulations regarding schools that are safe from disasters such as Regulation of the Minister of Education and Culture of the Republic of Indonesia No. 33 of 2019 concerning the Implementation of the Disaster Safe School Education Unit Program, which states that there is integration related to disaster management into the national curriculum and local content curriculum. Apart from that, the government has also issued Regulation of the Head of the National Agency for Disaster Management No. 4 of 2012 concerning the Implementation of Schools and/or Madrasas that is Safe from Disasters.

## Preparedness

### Knowledge

Knowledge and attitudes of individuals and households were seen based on surveys and interviews with several indicators such as the definition of a tsunami, the causes and signs of a tsunami and what to do when a tsunami occurs. This knowledge is the basis of preparedness. Knowledge of people living in disaster-prone areas is the main aspect in order to be aware of disasters that can be measured using indicators: disaster characteristics, early warning systems (EWSs), evacuation facilities such as shelters, evacuation maps and routes, experience and disaster simulations (Patrisina et al. 2018). According to Gregg et al. (2004), knowledge of the hazard will enable individuals to know about the risks that will be posed by the hazard. The results of the analysis show that the knowledge and attitudes of individuals and households in four villages towards tsunami disaster risk are in high category, and as many as 80% of households in four villages have a high category of knowledge and attitude towards tsunami disaster risk.

Knowledge of the tsunami disaster is the basis for emergency planning, resource mobilisation and early warning. With knowledge, individuals will know the right activities in anticipating a disaster. Based on the data, 85% of informants know the definition of tsunami. The knowledge about tsunami comes from individual and household experiences.

Experience of disaster events will provide knowledge to the community. Experience is something experienced in the past. Studies related to disaster experience state that there is a significant influence on preparedness. Experiencing a disaster event can lead to a higher perception of risk and will lead to individual preparedness plans (Wang & Zang 2018). Correspondingly, individuals who do not have disaster experience will tend to behave passively when there is an early warning (Walters, Mason & Ellis 2019). The signs of the 2004 tsunami disaster, such as an earthquake and receding seawater, provide knowledge so that the community can save themselves, likewise with the people in four villages, who on average knew about the tsunami because of the 2004 tsunami in Aceh. The experience of the tsunami disaster that had been experienced or seen made most of the informants recognise the characteristics of a tsunami event. This is evidenced by data showing that as many as 85% of informants know the signs of a tsunami.

### Policies and guidelines

The second parameter of preparedness in dealing with a tsunami disaster is policies and guidelines related to preparedness to anticipate a tsunami disaster. Regarding policies related to tsunami disaster management, only 22% of informants know about policies related to preparedness to tsunami disasters and only 17% of informants know guidelines for action plans in dealing with tsunami disasters. Based on observations, there has been no socialisation from the local government so that individuals do not yet know about policies and guidelines for tsunami preparedness. However, the plan for preparedness has been verbally agreed upon by the community. The community has agreed on an evacuation location, namely in the sub-district. If the waves get higher, then the evacuation site is in the Kuala Dua area. Kuala Dua is one of the villages in Muara Batu District, which is closest to the coast of Muara Batu District with a hilly topography:

‘All the people already know, if a tsunami disaster occurs, the community will go through the southern route, then to the sub-district office. If the wave height increases, the community will move up to the Kuala Dua area.’ (Participant 3, 43 years old, Village Head)

Other than policies and guidelines related to preparedness to anticipate a tsunami disaster, a very important regulation in disaster management is the availability of regulations based on disaster-prone areas. In Indonesia, especially in Muara Batu District, these regulations also exist, namely in the form of Qanun, although they have not been fully implemented. A study conducted by Ayuningtyas et al. (2021) showed that the implementation of regulations and policies in disaster management in Indonesia has not yet been optimal.

### Emergency response plan

The knowledge possessed by the individual and household must be followed by an emergency response plan. As many as 88% of informants have planned an emergency, which shows that individuals and households in four villages are ready with a plan for rescue during a disaster emergency. The knowledge of tsunami signs, evacuation sites and evacuation routes make people know how to save themselves when a tsunami disaster occurs. Disaster emergency plan needs to be developed in order to take proactive action in disaster preparation, which includes protecting assets, self and family evacuation, engagement with family, communication, supplies and live stocks (Patrisina et al. 2018).

As explained in the previous sub-chapter, the community knows where to evacuate during a tsunami disaster. In Muara Batu District, they only use the district office as an evacuation place, and if the water gets higher, residents evacuate to a higher village, namely Kuala Dua Village. In general, evacuation is divided into two, namely horizontally by moving away from the beach or vertically by heading towards the evacuation building (McCaughey et al. 2017). Determining a tsunami evacuation building as an optimal evacuation site needs to consider several things such as the characteristics of the tsunami arrival, road network and width, procedures of danger warnings and the time of occurrence. Apart from that, evacuation routes also need to be developed to avoid crowding during the evacuation process by paying attention to several parameters such as pedestrian speed, available routes, route width and number of people who need to be evacuated (Koshimura et al. 2006 & Wood et al. 2016). A study conducted by Soviana et al. (2023) in Banda Aceh City showed that the main road of Banda Aceh City consists of six lanes and two lanes, with lane widths capable of accommodating vehicles with a width of no more than 2.5 m. Secondary roads consist of two lanes and two lanes with road widths ranging from 4 m to 5 m.

However, almost all the informants did not prepare important documents and essential medicines, sufficient ready-to-eat and durable food, alternative means of communication, family photos, and addresses and/or phone numbers. Perry and Lindell (2008) said that preparing emergency equipment to anticipate disaster is one of the preparedness activities. The basic emergency equipment according to Federal Emergency Management Agency (FEMA 2020) refers to water, food, cash, battery-powered or hand crank radio, flashlight, and extra batteries, first aid kit, whistle, masks and local maps.

### Tsunami warning system

One of the parameters of preparedness is disaster warning system. Early warning system is an integrated system of hazard monitoring, forecasting and prediction, disaster risk assessment, communication and preparedness activities systems and processes that enables individuals, communities, governments, businesses and others to take timely action to reduce disaster risks in advance of hazardous events (UNISDR 2016). There are four components of EWSs, namely risk knowledge, monitoring and warning, dissemination and communication, and response capability (UNISDR 2006; WMO 2016). The EWS in four villages is still traditional, using sirens or loudspeakers from each mosque or *meunasah*. An EWS is very important because it serves as a marker for the community to immediately respond to disasters that occur. Based on the data, 70% of the informants answered that they knew the tsunami warning system. This is because this siren has become a hereditary thing, namely every time there is a siren it indicates that there is something important when there is a siren sound in the *meunasah*.

The EWS in the four villages has been applied. This was explained by each village chief and youth who said that when an earthquake occurred, there was a special task force or usually youths who were sent or voluntarily monitor the condition of the ocean. If there are signs of a tsunami, the youth will inform the village chief to notify the community to save themselves to a higher place. Based on the information obtained, the signs of a tsunami that are understood are first an earthquake followed by receding seawater. Dudley and Min (2006) explained that tsunamis are caused by earthquakes, landslides, volcanic eruptions or the fall of meteors that occur in the sea. The public understands that the signs of a tsunami are earthquakes. The public knowledge about the signs of a tsunami and the traditional system of early warning is not only as a result of experience but also because the community has received training from several foreign non-governmental organisations. The receding seawater shortly after the earthquake also served as a sign for the people of Simeulue, Aceh, to save themselves when a tsunami occurred. This is called as *smong* which is the local wisdom of the people of Simeulue, Aceh, who consider tsunamis to be *smong* and make *smong* into a tsunami EWS (Syafwina 2014; Rahman, Sakurai & Munadi 2017).

Currently in Indonesia, there is INA-TEWS, which is a tsunami disaster warning system. Tsunami EWS is based on an earthquake and tsunami observation network consisting of seismometers, geodetic sensors and sea level measurement stations, which then transmit real-time data to Tsunami Service Providers (TSPs) and National Tsunami Warning Centers (NTWCs) (UNESCO 2022). Tsunami EWS in Indonesia also applies the real-time global positioning system (GPS) deformation monitoring method or monitoring deformation fields installed along the coast of the Indian Ocean and information can be obtained 5 min–10 min after the earthquake and can be immediately used to detect the potential for a tsunami because of the earthquake. The seismicity data are then sent in real time via satellite to the BMKG warning centre in Jakarta. Tsunamis in Indonesia are included in the near-field category where the distance from the earthquake epicentre is approximately 200 km with a very short arrival time of the tsunami, namely 10 min to 1 h, so the main challenge in developing a tsunami EWS in Indonesia is how to know the character of the deformation zone formed by earthquake in detail accurately (Lauterjung, Münch & Rudloff 2010; BMKG 2012; Kurniasih, Marin & Setyawan 2020).

### Resource mobilisation

Based on data, only 7% of the community had received training in tsunami emergency response. The low parameter value indicates that the capacity of individuals and household in mobilising thir resources during tsunami disaster is still low. That is because the community has never received any preparedness training. Apart from the information from the community, no community has prepared all the things or needs such as disaster preparedness bags and important documents in one place so that when a tsunami occurs, they can be used or taken immediately. Although disaster preparedness varies in time, place and type of disaster, two common components in preparedness are preparing emergency supplies and creating an emergency plan (Lam et al. 2017). In Indonesia, emergency equipment is referred to as a disaster preparedness bag, which is a package of items for survival, such as clean water, food and first aid supplies, while an emergency plan refers to special procedures for dealing with sudden or unexpected situations (Bhanumurthy, Shankar, Rao & Nagamani 2015). The existence of a disaster preparedness bag will make it easier for people to evacuate when a disaster emergency occurs (Utariningsih et al. 2023).

### Individual and household preparedness

Based on the surveys and interviews, the preparedness of households in four villages in dealing with the tsunami disaster is in the ready category, which is 60.5%. Based on the analysis conducted, the individual and community of four villages has a high level of knowledge and attitude. Even though there are no guidelines or rules, knowledge and community emergency response plans are still high. This knowledge should be trained, or simulation to increase community preparedness in anticipating a tsunami disaster. Appropriate and effective community preparedness in dealing with disasters can be seen from the extent to which they can be responsive in responding to disasters, which is in accordance with the statement (Herdwiyanti & Sudaryono, 2012).

Disaster preparedness refers to the activities and actions taken to ensure an effective response to the impact of hazards (Paton 2019; Dasgupta et al. 2020). Increasing preparedness needs to be a special concern for high-risk areas such as Muara Batu District. Increasing preparedness can be performed by conducting socialisations and disaster simulations. This is as suggested by Julianto et al. (2019) and Utariningsih et al. (2022), where preparedness knowledge can increase after disaster socialisation is carried out.

### Government preparedness

Government preparedness related to relevant policies and guidelines is still lacking in all locations. The government is still concentrated on disaster management, while preparedness is not yet a focus. Organisations such as the Gampong youth organisation, fishermen’s groups and Family Welfare Programme or *Program Kesejahteraan Keluarga* (PKK) also explained that efforts to improve preparedness had never been carried out in the four locations. In addition to this, the village head explained that early warnings are usually used by sirens from the *meunasah*. The data obtained also explain that the Gampong government in all research locations also does not know about tsunami EWS. They use a tsunami sign, namely an earthquake. After there was an earthquake, the youths were assigned to shore up the beach. If the water recedes, the monitoring team will inform the village head to be conveyed to the community via loudspeakers in the *meunasah*.

Until now, Indonesia is still a country with earthquakes that can also cause tsunami disaster. Based on the Centre for Research on the Epidemiology of Disasters (CRED 2022), Indonesia is included in the 10 countries with the highest mortality in the world because of earthquake disasters (Table 1).

**TABLE 1:** Top 10 mortality from disaster.

Country	Disaster	Fatalities
Europe	Heat wave	16.305
Uganda	Drought	2.465
India	Flood	2.035
Afghanistan	Earhquake	1.739
Nigeria	Flood	603
South Africa	Flood	544
Philippines	Tropical storm	346
Indonesia	Earthquake	334
Brazil	Flood	272

Note: European heat waves in Spain, Germany, the UK, France, and Portugal were grouped into one event.

*Source*: Centre for Research on the Epidimiology of Disaster (CRED), 2022, *Disaster year in review 2022*, viewed from https://cred.be/sites/default/files/2022_EMDAT_report.pdf

In mobilising resources, the Gampong government has coordinated with other stakeholders such as the PKK, youth groups and fishing groups. This shows that the government’s preparedness is still quite high. Preparation of equipment for evacuation sites such as tents has also been provided by each village government. Apart from that, there is no equipment prepared by the village or sub-district government. The government’s readiness is closely related to the ongoing disaster experience.

## Conclusion

The government has built breakwaters and residents have planted coconut trees along the coast, namely on the coast of Gampong Tanoh Anoe, Cot Seurani and Meunasah Lhok. In addition, regulations for land use in disaster-prone zones already exist, but along the coast, which is a disaster-prone area, residents have used it as a place to sell and tourist attractions to residential areas. The implementation of education was carried out in the four villages studied in the post-tsunami disaster. Meanwhile, regarding the preparedness of the community in Muara Batu District in dealing with the tsunami disaster, both the community and the local government concluded that the preparedness of the community, both households and the government in Muara Batu District in dealing with the tsunami disaster, was classified as ready. This study recommends strengthening mitigation and preparedness with periodic community training by the village or district government. This will increase and strengthen synergies and interconnections between communities, village and district governments in dealing with the tsunami disaster.
